# Lower Frequency of Multiple Erythema Migrans Skin Lesions in Lyme Reinfections, Europe

**DOI:** 10.3201/eid3104.241329

**Published:** 2025-04

**Authors:** Franc Strle, Vera Maraspin, Stanka Lotrič-Furlan, Katarina Ogrinc, Tereza Rojko, Andrej Kastrin, Klemen Strle, Gary P. Wormser, Petra Bogovič

**Affiliations:** University Medical Centre Ljubljana, Ljubljana, Slovenia (F. Strle, V. Maraspin, S. Lotrič-Furlan, K. Ogrinc, T. Rojko, P. Bogovic); University of Ljubljana, Ljubljana (A. Kastrin); Tufts University School of Medicine, Boston, Massachusetts, USA (K. Strle); New York Medical College, Valhalla, New York, USA (G.P. Wormser).

**Keywords:** *Borrelia burgdorferi*, Lyme borreliosis, erythema migrans, bacteria, Lyme disease, tick-borne infections, ticks, vector-borne infections, clinically manifested reinfections, odds for disseminated infection, Europe

## Abstract

The erythema migrans (EM) skin lesion is the most common clinical manifestation of Lyme borreliosis. Information about EM in Lyme borreliosis reinfection is limited. Of the 12,384 cases with diagnosed EM at an outpatient clinic during 1990–2014 in Slovenia, 1,962 (15.8%) cases occurred in patients who were treated previously for Lyme borreliosis, including 1,849 (94.2%) who had previously had EM. The percentage of reinfected patients who sought care with disseminated Lyme borreliosis at the time of reinfection, as manifested by multiple EM skin lesions, was significantly lower than for EM patients with no history of Lyme borreliosis (5.5% [108/1,962] vs. 7.4% [769/10,427]; p = 0.002). None of the clinical manifestations of Lyme borreliosis in Europe will completely protect against EM developing in patients in the future. The reoccurrence of Lyme borreliosis manifested by multiple EM lesions is significantly less likely than for patients with no history of Lyme borreliosis.

Lyme borreliosis (LB) is the most common tickborne disease in the Northern Hemisphere. LB is caused by several species of Lyme borreliae (*Borrelia burgdorferi* sensu lato). LB in North America is caused almost exclusively by *B*. *burgdorferi* sensu stricto (hereafter referred to *B. burgdorferi*), whereas LB in Europe is mostly caused by *B*. *afzelii*, *B*. *garinii*, *B*. *bavariensis*, and *B. burgdorferi*. The clinical manifestations differ somewhat according to the infecting *Borrelia* species (*1*–*4*). However, regardless of the causative agent, the erythema migrans (EM) skin lesion is typically the initial and most common clinical manifestation of infection caused by the 4 *Borrelia* species listed. 

LB is usually treated successfully with antimicrobial drugs. In untreated patients with a single EM skin lesion, this lesion will typically disappear within a few weeks to a few months (*1*–*3*); however, manifestations of disseminated LB may still become apparent, such as development of additional EM skin lesions or of extracutaneous manifestations. Lyme neuroborreliosis, Lyme carditis, and Lyme arthritis are the most frequent extracutaneous clinical manifestations of LB in both Europe and North America. In Europe, 2 additional skin manifestations may occur: borrelial lymphocytoma, which occurs early in the course of infection, often in conjunction with EM; and acrodermatitis chronica atrophicans (ACA), a late, chronic skin lesion associated with an elevated IgG response to Lyme borreliae (*1*–*3*,*5*,*6*).

The pathogenesis of LB is not fully understood. *Borreliae* spp. enter the skin from the bite of an infected tick and are thought to disseminate from the skin to other anatomic sites through the blood stream. Lyme borreliae do not express any known toxins that cause disease. However, they contain a large array of lipoproteins that can trigger a robust host immune response, which is potentially useful for control of the infection (*2*). The site of infection and the host immune responses are thought to be key determinants of the clinical signs and symptoms of LB (*2*,*7*–*10*). The immune response might serve to protect against or modify the manifestations of reinfections.

In Europe, only ≈40% of Lyme borrelia infections are symptomatic (*11*–*15*), whereas in the United States, ≈90% of Lyme borrelia infections are symptomatic (*16*). However, information on the clinical manifestations because of symptomatic initial infections followed by symptomatic reinfections with *B. burgdorferi* s.l. is limited in both geographic areas. The available information is on the basis of several case reports (*17*–*28*) and on some relatively small case series consisting of up to 40 patients (*29*–*36*). In most of those published reports, the clinical manifestation of reinfection has been a solitary EM skin lesion, which was most often observed after a successfully treated previous infection that was usually a solitary EM skin lesion. Therefore, having had LB does not prevent the development of a subsequent solitary EM (localized infection), although in the United States it might prevent symptomatic reinfection because of the same genetic lineage of *B. burgdorferi* that caused the primary infection (*35*,*37*,*38*). Hypothetically, a prior disseminated infection might better protect against reinfection. Nevertheless, there have been a few case reports of symptomatic *Borrelia* spp. reinfection in patients who previously had Lyme neuroborreliosis (*19*,*23*,*27*), Lyme arthritis (*21*,*23*), and ACA (*39*,*40*).

The incidence of LB in Slovenia is among the highest of countries in Europe. Mandatory public health notification of symptomatic cases began in 1986. After an initial steady increase in the number of reported cases, the incidence rate has markedly fluctuated over the past 2 decades, reaching a peak in 2020 (390 cases/100,000 inhabitants) and a large predominance (>90%) of EM cases (*41*). The Lyme borreliosis outpatient clinic (LBOC) at the Department of Infectious Diseases of the University Medical Centre, Ljubljana, Slovenia, was established in 1988; the LBOC evaluates and treats patients with any of the potential clinical manifestations of LB. The >3 decades of LBOC operation, in conjunction with the high volume of patients and an organized collection of information, has provided insights into LB and provided information relevant to our study.

The objective of this study was to better understand reinfection with *B. burgdorferi* s.l. Specifically, we aimed to evaluate if reinfections clinically manifesting as EM occur only after EM or also after other manifestations of LB; if previous LB infection provides protection against disseminated disease (clinically manifested as multiple EM for this study); and if different manifestations of LB differ in providing protection against new episodes of disseminated disease (clinically manifested as multiple EM).

## Methods

Our primary source of information used in this study was the database of patients >15 years old with EM identified at the LBOC during 1990–2014. We obtained those data prospectively by using a questionnaire that did not change substantively during the 25-year period and included if LB had been diagnosed and treated before the current visit and, if yes, what were the clinical manifestations.

### Patients

To evaluate reinfections, we assessed patients ≥15 years of age, confirmed with EM at the LBOC during 1990–2014, for inclusion in this study. Inclusion criteria for participation were having >2 episodes of symptomatic *Borrelia* infection, with all reinfection cases having EM. For a subgroup of patients who reported having been confirmed with and previously treated for LB before attending the LBOC for EM, we obtained clinical documentation for verification of the previous confirmation. Because the available medical documentation of EM cases previously confirmed and treated by the patients’ primary physicians did not enable a reliable differentiation between solitary EM and multiple EM, we interpreted those cases as having had EM.

### Definitions

#### Clinically Manifested Primary Infections and Reinfections

LBOC patients with EM who claimed they never had LB previously and were not found in the LBOC database were interpreted to have initial EM. Patients at the examination at our LBOC for EM who claimed they were treated for LB previously elsewhere, or who were found in our database to have LB confirmed before the current visit at the LBOC, were interpreted to have EM because of reinfection.

#### Clinical Manifestations of LB

We defined EM as an expanding red or bluish-red skin lesion, with or without central clearing, that developed days to weeks after the bite of a tick or exposure to ticks in a LB endemic region and was >5 cm in diameter. We defined multiple EM lesions as the presence of >2 EM skin lesions, >1 fulfilling the size criteria for a solitary EM (*42*). To distinguish between relapse of EM versus EM occurring because of reinfection, the diagnosis of reinfection required that the initial EM skin lesion had to have completely disappeared after antimicrobial treatment (*35*) and that the new EM skin lesion had to have occurred at a different skin site. Furthermore, because in patients with ACA EM-like skin lesions may emerge from the border of an existing ACA skin lesion (*43*), to satisfy requirements for the diagnosis of a new EM, the EM had to occur at a site of a new tick bite and remote from the site of the ACA skin lesion.

We defined Lyme neuroborreliosis by all of the following characteristics: the presence of signs or symptoms suggestive of nervous system LB with no obvious other explanation; cerebrospinal fluid pleocytosis (>5 × 10^6^ leukocytes/L); and the demonstration of *Borrelia* infection by using intrathecal *Borrelia* spp. antibody production calculated according to Reiberˊs formula (*44*), or isolation of *Borreliae* spp. from a cerebrospinal fluid culture, or the presence of EM within 3 months before onset of the neurologic symptoms. Criteria for borrelial meningoradiculoneuritis (Bannwarth syndrome) were the same, except that an additional requirement was having radicular pain.

We defined ACA first by the clinical course and appearance of the skin lesions. Then, we also considered elevated serum levels of *Borrelia* spp. IgG and consistent histologic skin findings (*5*,*42*). 

We characterized Lyme arthritis by recurrent attacks or persistence of objective joint swelling in 1 or multiple large joints. In addition, we considered the presence of borrelial serum IgG and the exclusion of alternative explanations for the arthritis (*42*).

We defined borrelial lymphocytoma as a painless bluish-red nodule or plaque on the ear lobe, ear helix, nipple or scrotum, or very rarely on other parts of the body, with no other obvious explanation. Clinical suspicion was confirmed by the concomitant presence of EM or other manifestations of LB or *Borrelia* spp. antibodies in serum. In locations outside of the ear lobe and scrotum, histologic examination showing an intense polyclonal B-lymphocytic infiltration was required (*6*,*42*).

### Statistical Analyses

We summarized continuous variables by using medians and interquartile ranges and categorical variables as frequencies with percentages, accompanied by 95% CIs. We assessed associations between the categorical variables by using logistic regression models (Table), with the results reported as odds ratios (ORs), 95% CIs, and p values. To mitigate the risk for type I errors arising from multiple comparisons, we used the Benjamini-Hochberg procedure to adjust the p values, thus controlling the familywise error rate. We conducted all analyses by using the R Statistical Software package (The R Project for Statistical Computing, https://www.r-project.org).

**Table:** Erythema migrans diagnosed at the LBOC in Slovenia in relation to having had, or not having had, a previous episode of Lyme borreliosis*

EM diagnosed at LBOC, n = 12,384	No. cases	Years (IQR; range) before current EM	Solitary EM, n = 11,507	Multiple EM, n = 877	OR (95% CI)†	p value
No previous episode of LB: EM because of primary infection	10,422	NA	9,653	769		
Previous episode(s) of LB: EM because of reinfection	1,962	NA	1,854	108	0.73 (0.59–0.90)	0.002
Initial LB manifestation‡						
EM§	1,849	6 (3.5–8; <1–23)	1,745 (94.4; 93.2–95.4)	104 (5.6; 4.6–6.8)	0.75 (0.60–0.92)	0.005
Other well-defined manifestation¶	60	5 (3–6.5; 1–12)	59 (98.3; 91.1–99.9)	1 (1.7; 0.0–8.9)	0.21 (0.03–1.54)	0.12
LNB	37	4 (2.5–6)	36 (97.3; 85.8–99.9)	1 (2.7; 0.1–14.2)	0.35 (0.05–2.55)	0.30
LA	10	6 (4.5–7.5)	10 (100; 69.2–100)	0 (0; 0.0–30.9)	0.00 (0.00–∞)	>0.99
ACA	12	6.5 (4–8.0)	12 (100; 73.5–100)	0 (0; 0.0–26.5)	0.00 (0.00–∞)	>0.99
Borrelial lymphocytoma	1	2	1 (100; 25–100)	0 (0; 0.0–97.5)	0.00 (0.00–∞)	>0.99
Nonspecific symptoms in conjunction with presence of borrelial serum antibodies#	53**	4.5 (2.5–7.0)	50 (94.3; 84.3–98.8)	3 (5.7; 1.2–15.7)	0.75 (0.23–2.42)	0.64

*Values are no. (%; 95% CI) except as indicated. ACA, acrodermatitis chronica atrophicans; EM, erythema migrans; IQR, interquartile range; LA, Lyme arthritis; LB, Lyme borreliosis; LBOC, Lyme borreliosis outpatient clinic; LNB, Lyme neuroborreliosis; NA, not applicable. †In comparison to the ratio of multiple EM versus solitary EM in EM skin lesions because of primary infection (769/9653, 7.97%; OR 1). ‡If >1 manifestation, only the non-EM manifestation is shown (for example, in patients with EM and LNB, only LNB is tabulated. No patient had >1 non-EM manifestation). §Initial EM was diagnosed elsewhere (n = 1,102) or at the LBOC (n = 747). ¶The listed initial manifestations of LB were diagnosed at the LBOC. #Treated with antibiotics effective to treat LB for nonspecific symptoms (myalgia, arthralgia, headache, fatigue, etc,, but without an objective clinical manifestation of LB) in association with the presence of serum borrelial antibodies. **51 cases diagnosed elsewhere, 2 at the LBOC.

### Ethics

This study was conducted in accordance with the Declaration of Helsinki and was approved by the Medical Ethics Committee of the Republic of Slovenia (project identification code no. 0120–551/2023/3). The Ethics Committee waived the need for written informed consent.

## Results

During 1990–2014, a total of 12,384 cases of EM were diagnosed in 11,642 patients >15 years of age at the LBOC (Table). Of those cases, 15.8% (1,962) of patients indicated they were confirmed with and treated for a prior episode of LB either at the LBOC or by their primary care physician and 10,422 were a primary infection. The 1,962 reinfection cases of EM occurred in 1,855 patients; most (1,767) patients had 1 clinically manifested reinfection, 73 patients had 2 reinfections, 14 patients had 3 reinfections, and 1 patient had EM 7 times in addition to the initial infection. The duration of EM skin lesions before diagnosis at the LBOC in the reinfection group and for EM occurring because of a primary infection was comparable: median 9 (IQR 4–19) days versus 10 (IQR 4–24) days (p = 0.20). Furthermore, although the duration of EM before diagnosis at our LBOC was longer in patients with multiple EM skin lesions compared with those with a solitary skin lesion (median 13 [IQR 5–25] days vs. 10 [IQR 4–24] days), the difference was not significant (p = 0.07).

Of the 1,962 reinfections that manifested as EM, the most frequent clinical manifestation of the initial episode of LB was EM (94.2%, 1,849); however, there were also 37 cases of Lyme neuroborreliosis, 10 cases of Lyme arthritis, 12 cases of ACA, 1 patient with borrelial lymphocytoma, and 53 patients treated for nonspecific symptoms in conjunction with a positive test for borrelial serum antibodies. The median time interval from the primary infection to reinfection was ≈6 years (range 3 months–23 years) (Table). In 1,102 (59.6%) of the 1,849 cases with a previous EM and 51 (96.2%) of the 53 previous LB cases with nonspecific symptoms associated with a positive borrelial serology were tested at a clinical practice other than the LBOC, whereas the other prior LB cases, comprising all of the well-defined prior objective manifestations of LB other than EM, including Lyme neuroborreliosis, Lyme arthritis, ACA, or borrelial lymphocytoma, were tested and confirmed exclusively at the LBOC (Table).

The proportion of the EM cases with multiple EM skin lesions in the reinfection group was significantly lower than in the patient group who did not have a previous episode of LB (5.5% [95% CI 4.5%–6.5%], 108/1,962, vs. 7.4% [95% CI 6.9%–7.9%], 769/10,422; p = 0.002). In addition, within the subgroup of the 1,962 reinfection cases, the proportion with multiple EM was higher in cases with the first symptomatic reinfection than in subsequent reinfections (5.7% [95% CI 4.7%–6.9%], 101/1,767, vs. 3.6% [95% CI 1.5%–7.3%], 7/195); however, the difference was not statistically significant (p = 0.22). Furthermore, in the multiple EM subgroup, the number of skin lesions was lower in patients who previously had had LB, in comparison to those without a prior episode of LB. Of 108 patients with multiple EM who had LB previously, 42 patients (38.9% [95% CI 29.7%–48.8%]) had >3 EM skin lesions, whereas of the 769 patients with primary EM, 388 patients (50.5% [95% CI 46.9%–54.1%]) had >3 EM skin lesions (p = 0.02).

In the subgroup confirmed and treated for LB previously, the odds of seeking care with multiple EM ≈6 years after primary infection were on average 1.37× lower than in patients without prior LB (OR 0.73 [95% CI 0.59–0.90]). The odds were dependent on the previous manifestation of LB: the odds were 1.33× lower (OR 0.75 [95% CI 0.60–0.92]) when the previous manifestation was EM, 4.70× lower (OR 0.21 [95% CI 0.03–1.54]) when the previous manifestation was a well-defined other manifestation of LB (Lyme neuroborreliosis, Lyme arthritis, ACA, or BL), including Lyme neuroborreliosis (OR 0.35 [95% CI 0.05–2.55]), Lyme arthritis (OR 0 [95% CI 0.00–∞]), or ACA (OR 0 [95% CI 0.00–∞]), and 1.33× lower (OR 0.75 [95% CI: 0.23–2.42]) for the group of patients with nonspecific symptoms (myalgia, arthralgia, headache, fatigue) associated with the presence of borrelia IgG in serum. However, in view of the small numbers of cases in those subgroups, only the difference for having had EM previously was significant (Table). Of the 37 patients with Lyme neuroborreliosis who later sought care for a new episode of EM, only 1 (2.7%) had multiple EM, whereas 0/10 patients with Lyme arthritis, 0/12 with ACA, and 0/1 patients with borrelial lymphocytoma had multiple EM (Table).

## Discussion

Knowledge of the clinical manifestations of reinfection with Lyme borrelia is far from complete, and the factors that provide protection against clinically manifested reinfection with *Borrelia* spp. are not well understood. Immunologic factors and taking more precautions to avoid tick bites might play a role. It is assumed the presence of *Borrelia* spp. antibodies plays a partial protective role. Because disseminated borrelial infection and longer-lasting infection evoke a higher and more pronounced immune response than early localized infection, such as a solitary EM (*1*–*3*), it might be expected that protection against reinfection would be better after having had Lyme neuroborreliosis, Lyme arthritis, or ACA than after a solitary EM and that protection would be more effective against disseminated infection than against localized infection. However, humoral immunity would be expected to be temporal, meaning better protection in the first few years but less farther in the future. Furthermore, the variety of different *Borrelia* spp. that cause LB in Europe might negatively affect protective immunity against reinfection, because strain specificity in terms of protection was reported in the United States (*35*,*37*,*38*). Therefore, it seems reasonable there would be a higher risk for symptomatic reinfections in Europe, but we did not find data directly supporting this hypothesis in the published literature.

In previous reports, the clinical manifestation of reinfection has typically been a solitary EM skin lesion and was most often documented after a successfully treated previous infection, in which there was also a solitary EM skin lesion (*17**–**36*). However, hypothetically, a prior treated infection might better protect against a subsequent infection with clinical manifestations indicative of disseminated infection. The large number of patients with LB included in this study has provided useful information on reinfections with *B. burgdorferi*. Our findings show that EM may develop after diverse manifestations of LB, not only after a previous EM but also in patients who had Lyme neuroborreliosis, Lyme arthritis, or even ACA, a skin manifestation associated with a pronounced, protean, and long-lasting antibody response. A PubMed literature search revealed very few reports on individual cases of EM that occurred in patients who were previously successfully treated for Lyme neuroborreliosis (*19*,*23*,*27*), a mention of 2 patients previously treated for ACA in whom EM developed (*39*,*40*), and 3 cases of EM after Lyme arthritis (*21*,*23*). All those cases were from Europe, except for a report of 2 children in the United States in whom EM developed after having had Lyme arthritis (*21*). However, Lyme arthritis is more common in North America than in Europe.

Our study indicates that having had LB in the past does not provide complete protection against developing symptomatic reinfection (particularly developing a solitary EM) or against developing disseminated disease (defined in this study as having multiple EM). However, our results suggest that at a median time of ≈6 years after the initial episode of EM in Europe the likelihood of developing multiple EM is significantly lower than in EM patients with no known previous LB (1.3× lower; p = 0.005), whereas in patients who had an extracutaneous manifestation of LB or had ACA, the chance of developing reinfection manifesting as multiple EM was nearly 5× lower. However, the latter difference was not statistically significant. Of note, despite the relatively large number of patients with EM because of *Borrelia* reinfection in this study (1,962 episodes of EM in 1,855 patients), a statistical assessment of the effect of the different clinical manifestations of a previous infection on the relative incidence of subsequent disseminated disease was limited by the relatively small number of such cases, because most of the primary infections were EM.

An alternative hypothesis for the lower proportion of multiple EM in the group of patients with EM as the manifestation of reinfection could be a shorter duration of EM before diagnosis and treatment than when EM occurred because of primary infection, because the appearance of multiple EM takes time. Our results do not confirm this hypothesis, however, because we did not find significant differences in the duration of the EM skin lesions between the 2 groups.

For primary EM episodes confirmed elsewhere (by the patients’ primary care family physicians), we had no reliable data on whether they had solitary or multiple EM, nor did we have data on the presence or level of serum borrelial antibodies in these patients. Therefore, it can only be hypothesized on the basis of previous studies (*45*) that approximately half of the EM patients had detectable borrelial antibodies at the time of the previous infection. For patients with other manifestations of LB, it was known they were IgG seropositive at the time of diagnosis of the primary LB episode, irrespective of where they sought care.

The design of our study enabled an evaluation of the extent that having LB previously protects against subsequently developing disseminated LB but did not enable an evaluation of protection against developing a solitary EM or assessment of whether protection against disseminated infection decreases with longer time periods after the primary infection. In addition, because only patients >15 years of age were included in this study, the results may not be applicable to younger adolescents and children.

Although our findings are representative of Europe, they may not be applicable to North America, where LB is nearly exclusively caused by *B*. *burgdorferi*, rather than by *B*. *afzelii*, *B*. *garinii*, or *B*. *bavariensis*, which are the *Borrelia* spp. most commonly responsible for LB in Europe (*1*–*4*,*46*). In addition, the immune response to infection with *B*. *burgdorferi* in North America seems to be greater than the immune response to infection with *B*. *afzelii* and *B*. *garinii* and even *B. burgdorferi* (*47*–*49*) from Europe. Also relevant, strains of *B*. *burgdorferi* from Europe and the United States represent distinct clonal lineages, which vary in virulence and inflammatory potential (*49*).

In conclusion, according to findings in patients from Slovenia >15 years of age who were confirmed with and treated for a previous episode of LB, a new episode of EM may develop not only in patients who previously had EM (1,849 cases), but also in those who had borrelial lymphocytoma (1 case), Lyme neuroborreliosis (37 cases), Lyme arthritis (10 cases), and ACA (12 cases), indicating that none of those clinical manifestations of LB in Europe completely protected against the future development of EM, the most common clinical manifestation of *B. burgdorferi* infection. Our study also showed that having had LB reduces the likelihood of subsequently developing disseminated reinfection. In addition, our study findings suggest that protection against subsequent development of multiple EM after primary infection may depend upon clinical manifestation of the primary infection. Prior LB occurring with EM or nonspecific symptoms provides lesser protection against reinfection, whereas having had other well-defined clinical manifestations of LB, including Lyme neuroborreliosis, Lyme arthritis, or ACA, provides greater protection.
